# A machine-learning capillary electrophoresis-based pattern to identify suspected sickle cell trait and α-thalassemia co-inheritance

**DOI:** 10.1515/almed-2026-0045

**Published:** 2026-07-01

**Authors:** David Ceacero-Marín, Anna Cortés Bosch de Basea, Isabel Puig-Pey Comas, Mónica Vidal Pla, Lourdes Sánchez Navarro

**Affiliations:** Clinical Laboratory, Bellvitge University Hospital-IDIBELL, Hospitalet de Llobregat, Barcelona, Spain

**Keywords:** alpha-thalassemia, anemia, sickle cell, sickle cell trait, thalassemia

## Abstract

**Objectives:**

Sickle cell trait (HbAS) and α-thalassemia are common conditions. Although their hematological laboratory findings have been extensively characterized individually, there is limited evidence on the hematological pattern when they are co-inherited. Early and simple diagnosis of HbAS/α-thalassemia allows for optimization of the clinical approach and provision of appropriate genetic counselling. The objective is to identify, using machine learning algorithms, the most relevant hematological quantities and to establish a useful cut-off value for screening patients with suspected HbAS/α-thalassemia.

**Methods:**

A database containing 235 patients carrying HbAS was obtained from the laboratory information system. The following variables were collected: hemoglobin and erythrocytes concentration, mean corpuscular volume (MCV), and the fractions of hemoglobin S (%HbS), A (%HbA), A2 and F. A multivariate clustering analysis was performed using the k-means algorithm to divide the dataset into two subgroups: HbAS and suspected HbAS/α-thalassemia. ROC curve was constructed for each variable to identify patients with suspected HbAS/α-thalassemia.

**Results:**

Patients with %HbS <37.6 % and %HbA >59 % were classified as suspected HbAS/α-thalassemia with 100 % sensitivity, 97.7 % specificity and AUC [95 % CI] of 0.989 [0.977–0.997]. Patients with suspected HbAS/α-thalassemia exhibited significantly lower hemoglobin concentrations (p=0.008), MCV (p<0.001) and %HbS (p<0.001). In contrast, significantly higher erythrocyte concentrations and %HbA (p<0.001) were observed, compared to HbAS alone.

**Conclusions:**

The proposed discriminant values enable suspected cases of HbAS/α-thalassemia to be identified. This facilitates the prioritization of genetic testing for α-thalassemia to be conducted, the provision of genetic and family counselling, and the risk to be accurately assessed for these patients.

## Introduction

Hemoglobinopathies are by far the most common monogenic disorders in the world [1]. This is due to natural selection, consanguineous marriages in certain countries, and improved health standards that have enabled longer lifespans [2].

Sickle cell anemia is an inherited hemolytic disease whose clinical manifestations vary widely depending on the genotype involved. The homozygous form (HbSS) is the most severe, while the heterozygous form (HbAS) do not present symptoms or health problems. However, in extreme environmental conditions, individuals with HbAS (sickle cell trait) may experience complications resembling those of HbSS. These patients with HbAS may suffer from thrombosis, hematuria and papillary necrosis [1], bone infarction [3], rhabdomyolysis and death following intense physical exertion or dehydration. Furthermore, there is evidence to suggest an association with thromboembolism and obstetric complications [2], 4].

The prevalence of HbSS is around 4 % in African countries like Nigeria [5] and around 0.08 % in France [6]. In contrast, the prevalence of the genetic predisposition to HbAS in tropical African countries like Nigeria or Angola is around 21 % [5], 7], 4 % in Brazil [8], 3.4 % in France [6] and 0.2 % in Mediterranean populations such as Cyprus [9].

Various studies have evaluated the impact of sickle cell trait on blood count values. Most of these studies have shown that HbAS has no impact on hemoglobin concentrations [10]. Similarly, most studies have shown no effect on mean corpuscular volume (MCV). However, some studies suggest that it may cause a slight reduction [11].

In contrast, double heterozygosity (the occurrence of HbAS alongside other hemoglobin variants, such as HbC or HbE, or different forms of thalassemia) creates a diverse clinical spectrum and a different hematological pattern. Specifically, the co-occurrence of HbAS with thalassemia in its various forms can significantly affect disease progression.

It is essential to accurately differentiate between patients who are carriers of HbAS in heterozygosity and those with double heterozygosity, as each group exhibits specific complication risks.

Alpha (α) thalassemia, when considered in isolation, is characterized by a reduction in the synthesis of α chains, which results in microcytosis and hypochromia, as indicated by a decrease in MCV and mean corpuscular hemoglobin (MCH) [12], 13].

The prevalence of α-thalassemia traits varies depending on the population, being particularly high in malaria-endemic areas of Africa. In African countries, it is estimated to be around 39–40 % [14], 15].

By contrast, the estimated prevalence in South American countries such as Brazil [16] and Mediterranean European countries such as Greece [17] and Italy [18] is around 6–8 %.

Although there is an abundance of data on HbAS and α-thalassemia in isolation, the systematic characterization of their combined effect is limited.

Co-inheritance is a public health concern in regions where both traits are prevalent. It is of particular epidemiological interest due to its potential relationship with protection against malaria [19]. Although information on its global distribution and frequency remains incomplete, the health burden is expected to increase due to a combination of natural selection, consanguinity in certain countries, and epidemiological transition. Additionally, globalization and migration from endemic areas are spreading these hemoglobinopathies to non-endemic regions. Therefore, clinical and laboratory services must anticipate and develop algorithms that consider co-inheritance to prevent diagnostic errors and optimize care [20].

In clinical practice, the initial screening for hemoglobinopathies uses protein-based techniques, such as electrophoresis and chromatography. The results enable the identification of patients who are eligible for genetic testing to confirm and stratify the diagnosis of thalassemia [21].

Identifying patients with HbAS and α-thalassemia is clinically relevant because it can lead to misinterpretation of microcytosis or low corpuscular indices. This could result in underdiagnosis of α-thalassemia, overdiagnosis of iron deficiency, or overestimation of the severity of sickle cell trait.

In this context, an early and simple diagnosis of patients with HbAS and α-thalassemia is essential for optimizing clinical management, providing appropriate genetic counselling, accurately assessing individualized risk, and improving the prognosis for each patient.

The objective of this study is to identify, using machine learning algorithms, the most relevant hematological parameters and to establish a useful cut-off value for screening patients with suspected co-inheritance of HbAS and α-thalassemia.

## Materials and methods

### Design of the study

A database containing information on 235 patients with HbAS was obtained from the laboratory’s computer system (Modulab, Werfen, Barcelona, Spain) between January 2025 and December 2025.

The hematological variables collected were: hemoglobin concentration, erythrocyte concentration, and MCV. These quantities were measured using the Sysmex XN-10 analyser (Sysmex^®^, Kobe, Japan). Hemoglobin and erythrocyte concentrations were measured using colorimetric and impedance methods, respectively.

Additionally, hemoglobin A, A2, F and S fractions (%HbA, %HbA2, %HbF and %HbS, respectively) were measured by capillary electrophoresis using a Capillarys 3-Tera analyser (Sebia, Barcelona, Spain).

In addition, several phenotypic variables were categorized as follows: anemia, when hemoglobin concentrations were below the lower reference limit (LRL); microcytosis, when MCV values were below the LRL; anemia with microcytosis, when both hemoglobin and MCV were below the LRL; and erythrocytosis, when erythrocyte concentrations were above the upper reference limit.

Throughout the study period internal and external quality control material were performed to verify the performance of the overall procedure and the instrument under use and to ensure the validity of the analytical phase.

Subjects with iron deficiency anemia, defined as a ferritin or iron serum concentration below the LRL, were excluded. Ferritin and iron concentrations were determined using an immunoturbidimetry assay on the Cobas c701 analyser (Roche Diagnostics^®^, Mannheim, Germany).

The study protocol had previously been approved by the Medical Research Ethics Committee of the hospital (PR082/25).

### Statistical analysis

An unsupervised machine learning analysis was performed to identify a pattern compatible with HbAS and α-thalassemia based on hematological parameters and hemoglobin fractions measured by capillary electrophoresis.

Initially, the parameters were standardized using z-scores. A multivariate clustering analysis was performed using the k-means algorithm, which classifies individuals into “k” clusters. The algorithmic process is designed to optimize the similarity between data within each cluster, while simultaneously ensuring the maximum possible separation of clusters based on the pattern of the variables included.

The optimal number of clusters (k) was selected using Silhouette width and corroborated with the Calinski–Harabasz index. The Silhouette index captures within-cluster cohesion and between-cluster separation, whereas the Calinski–Harabasz criterion reflects the ratio of between-cluster to within-cluster dispersion.

Principal component analysis (PCA) was performed on the previously standardized variables (z-score) in order to reduce dimensionality and facilitate visualization of the multivariate structure of the dataset. This transforms the 7-dimensional (7D) structure of the data into 2 dimensions (2D). The first two principal components (PC1 and PC2), which concentrate the largest proportion of the explained variance of the dataset, were used to project the observations onto a 2D plane (PC1–PC2). The centroids of the clusters obtained using k-means (the midpoint of each group) were added to this graph to visualize their location and the degree of separation between clusters.

After separating the data into subgroups using multivariate cluster analysis, variables between groups were compared. Categorical variables were compared using the χ^2^ test. Quantitative variables were analysed using Student’s *t*-test or Mann-Whitney U test, depending on the assumption of normality. A significance level of p-value <0.05 was established.

Ultimately, a receiver operating characteristic (ROC) curve was constructed for each variable in order to discriminate between both clusters and identify patients with suspected HbAS and α-thalassemia. The optimal cut-off point was estimated using the Youden index for each quantity, in order to maximize the combination of sensitivity and specificity to discriminate between both groups.

To internally validate the established cut-off point to discriminate patients with suspected HbAS/α-thalassemia co-inheritance, two resampling strategies were applied. First, repeated stratified 5-fold cross-validation was performed 200 times by randomly dividing the cohort into five approximately class-balanced subsets and evaluating the established cut-off point in the corresponding validation fold at each iteration. Second, bootstrap validation was performed using 2,000 bootstrap samples from the original cohort, and the predefined rule was evaluated in the patients not selected in each bootstrap sample. In both approaches, sensitivity, specificity, positive predictive value (PPV), and negative predictive value (NPV), were estimated.

In addition, an exploratory molecular validation subset was analysed, including HbAS carriers in whom molecular testing for α-thalassemia had been requested during routine clinical care according to the clinical course. This cohort comprised 10 patients, including 7 with a positive molecular result for α-thalassemia and 3 with a negative result. This subset was employed to assess the concordance between the predefined combined rule and the molecular diagnosis.

## Results

After excluding subjects who did not meet the inclusion criteria (subjects with iron deficiency aaemia), the total dataset (n=218; 88 (40.4 %) male and 130 (59.6 %) female) was partitioned into two clusters.

The optimal number of clusters (k) was selected by comparing internal validation metrics across candidate solutions (k=2–5), prioritizing the average Silhouette width and the Calinski–Harabasz index. Both indices reached their maximum at k=2 (Silhouette=0.389; Calinski–Harabasz=82.68).

The Silhouette index for k=3 was 0.224. The Calinski-Harabasz index for k=3 showed a value of 70.52.

**Figure 1:**
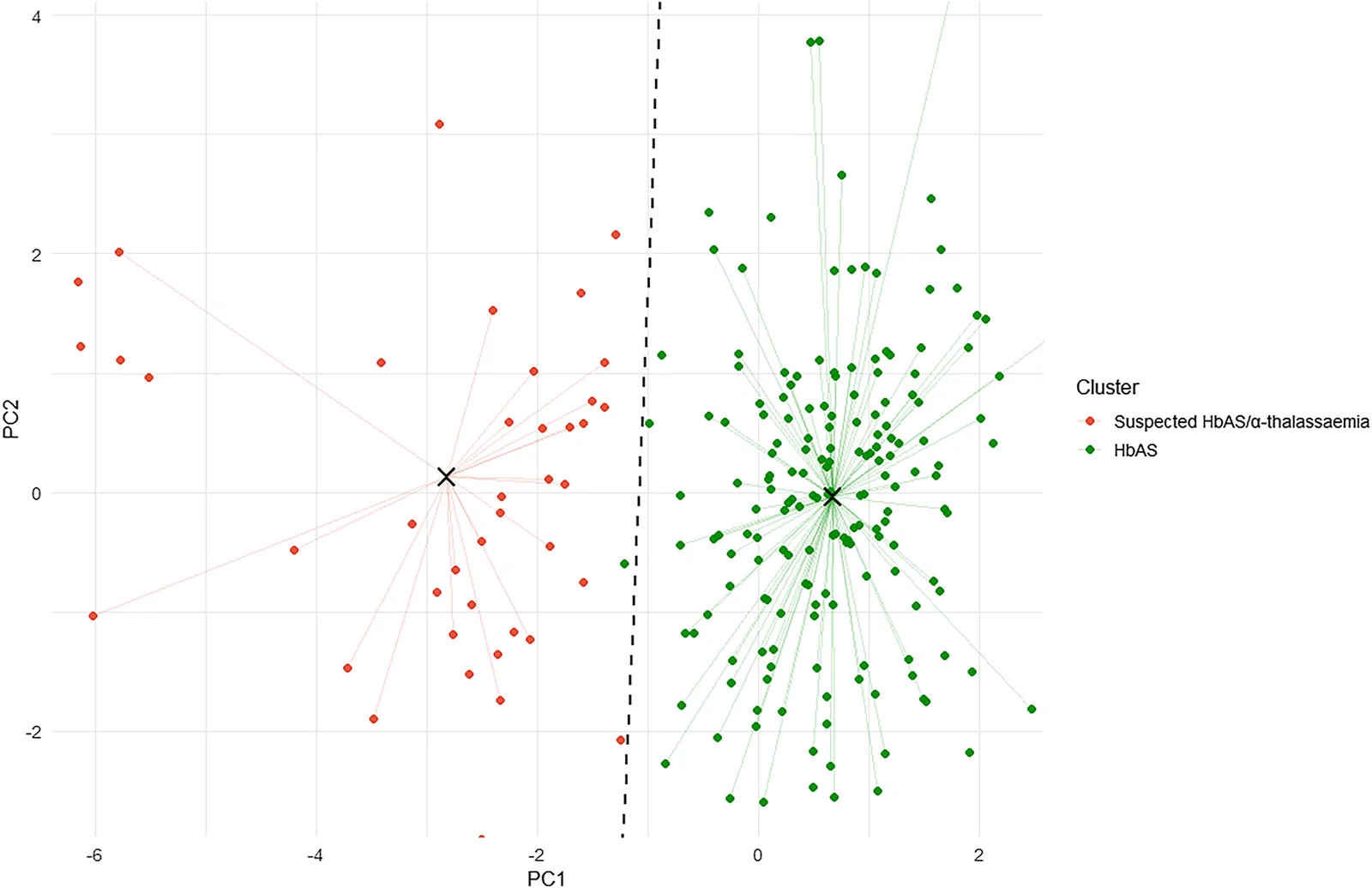
PCA projection (7 variables) with centroids, “vectors”, and equidistance boundaries. The figure shows the projection of each individual points on the plane defined by the first two principal components (PC1 and PC2) calculated from the seven standardized variables (z-score). The colors indicate the cluster assigned by k-means. The black crosses represent the centroids of each cluster in the PC1–PC2 space (mean of the PC1 and PC2 data of the subjects in each group). The thin lines extending from the points to their centroid illustrate the direction and magnitude of the displacement of each observation relative to the center of its cluster, providing a visualization of intragroup cohesion. The vertical dashed line corresponds to a decision boundary, defined as the geometric locus of equidistant points from both centroids; therefore, it separates the regions closest to the centroid of the HbAS cluster (right) and that of the cluster suspected of HbAS/α-thalassemia (left).

After partitioning the data into two groups, PCA was performed with seven standardized variables. The projection of individuals on the PC1–PC2 plane showed a clear separation of patients into two clusters (Figure 1). It should be noted that this figure is a 2D graphical representation, while cluster assignment was performed in the complete multivariate space (7D).

**Figure 2:**
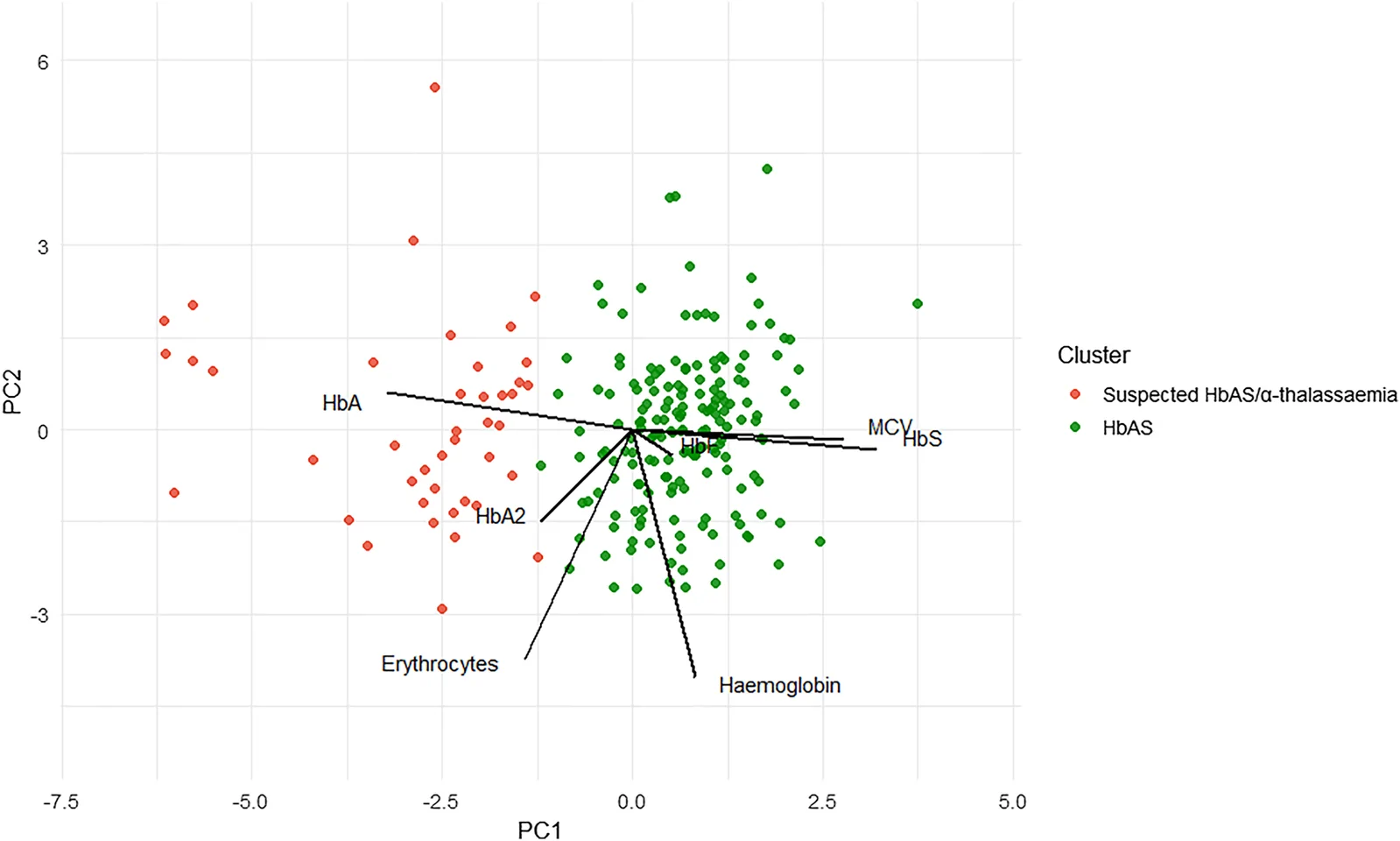
Biplot of principal component analysis (PCA) with 7 variables. Each point represents an individual projected onto the plane defined by the first and second principal components (PC1 and PC2), calculated from the standardized variables (z-score). The colours indicate the cluster assigned by k-means. The arrows correspond to the loadings of each variable and indicate the direction of increase of that variable in the PC1–PC2 space; their length is proportional to the contribution of the variable to the variability explained by these two components. Arrows pointing in similar directions indicate positively correlated variables, while opposite arrows suggest negative correlation. The separation between groups summarizes the multivariate differences between clusters.

The PCA biplot (Figure 2) allowed us to interpret the contribution of the variables to this separation. The suspected HbAS/α-thalassemia cluster was oriented toward a profile characterized by lower %HbS and MCV and, in contrast, higher %HbA and erythrocyte concentration. The orientation and length of the loadings suggested a minor contribution of %HbF to the overall pattern of separation in the PC1–PC2 plane.

**Figure 3:**
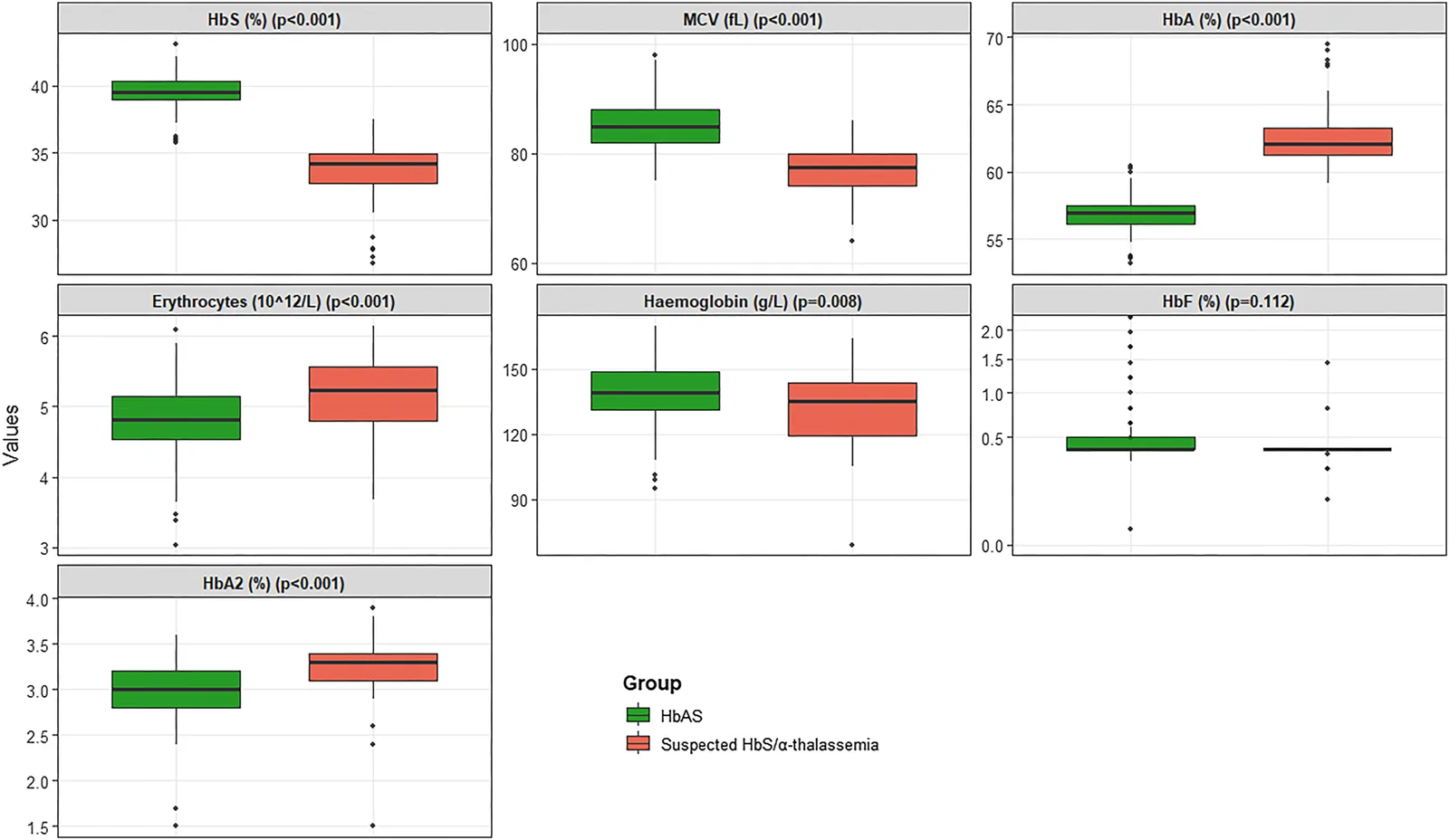
Box plot diagram of variables in the different groups.

Cluster 1 showed a predominantly microcytic phenotype, characterized by lower MCV (p<0.001), higher erythrocyte count (p<0.001), lower %HbS (p<0.001), and higher %HbA (p<0.001) than Cluster 2 (Table 1, Figure 3). This group also showed a markedly higher prevalence of microcytosis (p<0.001) and erythrocytosis (p=0.001), further supporting a pattern compatible with co-inheritance of sickle cell trait and α-thalassemia. By contrast, Cluster 2 displayed close to reference values red-cell indices and the expected %HbA/%HbS distribution for sickle cell trait, which is consistent with HbAS alone. Accordingly, Cluster 1 was interpreted as a suspected HbAS/α-thalassemia group, whereas Cluster 2 was considered consistent with HbAS alone.

**Table 1:** Comparison of variables between both groups.

Variables, units	Suspected HbAS/α-thalassemia (n=42)	HbAS (n=176)	p-Value
Age, years	43 (39–51)	51 (38–62)	0.027
Sex, male	20 (47.6 %)	68 (38.6 %)	0.373
Hemoglobin, g/L	135 (119–144)	139 (131–149)	0.008
Erythrocytes, ×10^12^/L	5.17 (0.54)	4.82 (0.50)	<0.001
MCV, fL	77 (74–80)	85 (82–88)	<0.001
%HbS, %	34.2 (32.7–34.9)	39.6 (39.0–40.3)	<0.001
%HbA, %	62.1 (61.2–63.3)	56.9 (56.1–57.5)	<0.001
%HbA2, %	3.3 (3.1–3.4)	3.0 (2.8–3.2)	<0.001
%HbF, %	0.4 (0.4–0.4)	0.4 (0.4–0.5)	0.112
Iron, µmol/L	15.0 (10.5–18.0)	16.5 (13.3–19.8)	0.320
Ferritin, µg/L	106 (43–191)	100 (55–211)	0.656
Anemia	11 (26.2 %)	32 (18.2 %)	0.339
Erythrocytosis	17 (40.5 %)	32 (18.2 %)	0.004
Microcytosis	39 (92.9 %)	57 (32.4 %)	<0.001
Anemia and microcytosis	10 (23.8 %)	10 (5.7 %)	0.001

Categorical variable is expressed as number (percentage). Quantitative data are expressed as mean (standard deviation) or median (interquartile range) depending on the normal distribution. %HbS, percentage of hemoglobin S; %HbA, percentage of hemoglobin A; %HbA2, percentage of hemoglobin A2; %HbF, percentage of hemoglobin F; MCV, mean corpuscular volume.

**Figure 4:**
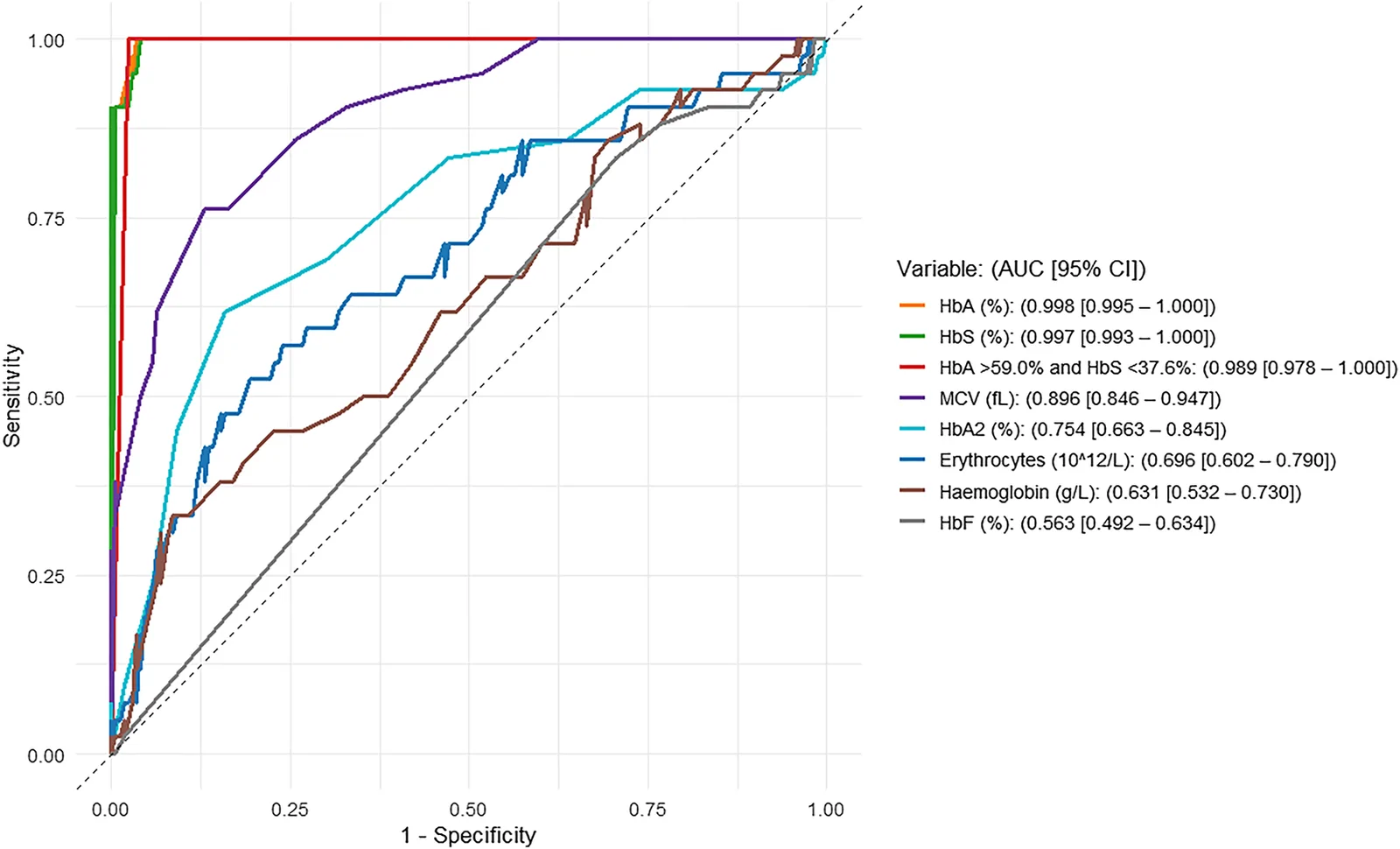
Receiver operating characteristic (ROC) curve for each variable to discriminate and identify patients with suspected HbAS and α-thalassemia.

The groups obtained (HbAS and suspected HbAS/α-thalassemia) after clustering analysis were used as reference groups for ROC analysis to obtain discriminatory thresholds for the different variables to identify suspected HbAS/α-thalassemia (Table 2, Figure 4).

**Table 2:** Cut-off point and rule of the different variables to discriminate and identify patients with HbS and α-thalassemia. PPV and NPV were obtained using the observed prevalence of cluster 1 in this cohort (19.3 %).

Variable	Proposed cut off	AUC [95 % CI]	Sensitivity, %	Specificity, %	PPV, %	NPV, %
%HbA	>59 %	0.998 [0.995–1.000]	100	96.6	87.5	100
%HbS	<37.6 %	0.997 [0.993–1.000]	100	96	85.7	100
MCV	<80.5 fL	0.896 [0.846–0.947]	76.2	86.9	58.2	93.9
%HbA2	>3.3 %	0.754 [0.663–0.845]	61.9	84.1	48.1	90.2
Erythrocytes	>5.15 10^12^/L	0.696 [0.602–0.790]	57.1	76.1	36.4	88.2
Hemoglobin	<123.5 g/L	0.631 [0.532–0.730]	33.3	91.5	48.3	85.2
%HbF	<0.45 %	0.563 [0.492–0.634]	83.3	29.5	22	88.1

AUC, area under the curve; 95 % CI, 95 % confidence interval; PPV, positive predictive value; NPV, negative predictive value.

Using a combined rule, %HbA >59 % and %HbS <37.6 % identified the suspected HbAS/α-thalassemia cluster with 100 % sensitivity, 97.7 % specificity, 89.4 % PPV 89.4 and 100 % NPV. AUC [95 % CI] was 0.989 [0.977–0.997] (Figure 4).

The predefined combined rule (%HbA >59 % and %HbS <37.6 %) showed excellent performance in the internal validation, confirming the stability of these findings. In the 5-fold cross-validation, the sensitivity and NPV remained 100 %, specificity was 97.1 % and PPV of 90.0 %. Similarly, bootstrap validation showed a sensitivity and negative predictive value of 100 %, with a specificity of 98.3 % and PPV of 92.3 %.

In the exploratory molecular confirmation subset, all patients with a positive molecular result for α-thalassemia (n=7) fulfilled the predefined combined rule (%HbA >59 % and %HbS <37.6 %) and were therefore classified into the suspected HbAS/α-thalassemia cluster. In contrast, patients with a negative molecular result (n=3) did not meet this rule and were classified into the HbAS cluster (Supplementary Table 1). These findings support the biological plausibility of the analytical classification and its concordance with molecular results in this subset.

## Discussion

This study introduces a novel approach to propose a cut-off point for %HbS and %HbA to identify individuals suspected of having a combination of HbAS and α-thalassemia.

The integration of a quantitative threshold of %HbS and %HbA as a pregenetic filter in diagnostic algorithms transforms an accessible, low-cost measurement into a clinically applicable screening tool that is aligned with daily healthcare practices. This has the effect of reducing diagnostic delays, associated costs, and unnecessary tests, and promoting accurate, individualized patient management.

The study highlights the implementation of machine learning tools within the laboratory’s computer system can be achieved through the integration of a centroid classifier, utilizing the Nearest Centroid Classifier algorithm. This tool automatically assigns each new patient to the nearest cluster in the standardized multivariate space. Consequently, these suspected patients can be identified, genetic testing for alpha thalassemia can be targeted, and genetic and family counselling can be optimized, especially for couples at risk.

The number of clusters was determined using Silhouette index and confirmed by Calinski–Harabasz criterion.

The Silhouette index reached its maximum value at k=2, which indicates an optimal balance between intra-cluster cohesion and inter-cluster separation. The Silhouette index value of 0.389 indicates adequate separation and cohesion between the groups with two clusters. Forcing k≥3 resulted in a decrease in the Silhouette index, suggesting groups that were closer to each other and less compact.

The Calinski–Harabasz criterion demonstrated its maximum value of 82.68 with k=2, which was not exceeded with k=3 (70.52) or k=4 (70.87). This finding lends further support to the hypothesis that a two-partition model provides a superior variance ratio between and within clusters. The findings of these indices indicate that the optimal balance between intracluster cohesion and intercluster separation, as well as the best differentiation between groups, was achieved with k=2.

Conventionally, the detection of hemoglobinopathies has relied on the utilization of high-performance liquid chromatography (HPLC). However, the implementation of capillary electrophoresis has led to significant advancements in the diagnosis of hemoglobinopathies, primarily through enhanced peak separation and reduced interference.

The available literature, based on HPLC, has shown a consistent decrease in %HbS when HbAS and α-thalassemia coexist. This phenomenon has been explained by a reduction in α-chain synthesis and a relative preference for α-chain assembly with βA over βS. This shift in the balance of hemoglobin formation leads to what is termed “peak inversion”, with an increase in HbA at the expense of HbS [22]. In this context, discriminating values for %HbS of 35 % in HPLC have been proposed as an indirect marker for suspected HbAS and associated α-thalassemia, especially when microcytosis coexists [23].

In contrast, no study has proposed cut-off points in capillary electrophoresis to identify patients with HbAS/α-thalassemia.

On this basis, and assuming that capillary electrophoresis reproduces similar quantitative behaviour, this study proposes a cut-off point in capillary electrophoresis of %HbS and %HbA. Consequently, patients exhibiting %HbS levels below 37.6 % and %HbA levels above 59 % are strongly suspected of having α-thalassemia in conjunction with HbAS.

The HbAS/α-thalassemia group demonstrated a statistically significant decrease in the median percentage of HbS, from 39.6 % in HbAS to 34.2 % in HbAS/α-thalassemia. In contrast, the HbAS/α-thalassemia group exhibited a substantial increase in the median percentage of HbA from 56.9 % in HbAS to 62.1 % in the HbAS/α-thalassemia group. These results reproduce the relative HbA/HbS “reversal” in practice.

These observations are comparable to those described in studies published by capillary electrophoresis, such as that of Mombo et al., which documented a distribution of %HbS in HbAS between 32 and 37 % (mean 35.2 %) [24].

A similar observation was made in a study that utilized HPLC, which revealed that patients with HbAS exhibited a mean percentage of HbS of 38.54 and 36.54 % in cases of HbAS/α-thalassemia. The mean HbA in HbAS was 56.57 %, and 58.81 % in HbAS/α-thalassemia [25].

Furthermore, the present study observed hematimetric changes consistent with the thalassemic pattern, including a reduction in MCV and an increase in erythrocyte concentration, consistent with findings reported in other studies such as Velasco-Rodríguez et al. [26].

The proposed rule showed consistent performance in the internal validation procedures, supporting its robustness and stability within the study sample and suggesting limited overfitting.

A limitation of the present study is that the classification of patients with thalassemic trait was not based on molecular analysis confirmation of the genetic mutations responsible for thalassemia, which are considered the gold standard for diagnosis. This omission stems from the lack of systematic genetic testing in routine clinical practice, attributed to high costs and limited availability across healthcare centres. This is especially when hematimetric and electrophoretic findings provide sufficient guidance for suspected diagnosis, as these methods are simpler and more cost-effective [27]. Additionally, informed consent is required to perform these tests. However, in order to provide further biological confirmation, a validation of the model was carried out on a small cohort of patients for whom molecular testing for α-thalassaemia had been requested as part of routine clinical practice. Complete agreement was observed between the classification provided by the predefined rule and the molecular result in this subsample, reinforcing the coherence of the analytical findings with the underlying molecular background. Nevertheless, these findings should be interpreted with caution, and confirmation in larger independent cohorts with broader molecular characterization will be necessary to establish the generalisability of this approach.

This study puts forward a new approach by suggesting a threshold for %HbS and %HbA with the aim of streamlining the identification of suspected cases of co-inheritance of HbAS and α-thalassemia. However, future validation studies comparing these subgroups using genetic analysis will be necessary to confirm the diagnostic accuracy of the proposed threshold.

## Conclusions

The proposed threshold for %HbS and %HbA, measured by capillary electrophoresis appears to be a useful tool for guiding the study of suspected cases of HbAS and α-thalassemia. This approach is of particular benefit when guiding genetic testing for α-thalassemia, offering genetic and family counselling, and accurately stratifying risk for these patients. Furthermore, it streamlines workflows and reduces time, costs, and the healthcare burden.

## Supplementary Material

Supplementary Material
