# Supplementary material for: A machine-learning capillary electrophoresis-based pattern to identify suspected sickle cell trait and α-thalassemia co-inheritance
Source: Adv Lab Med. 2026 Jul 1;7(3):202–10. doi: 10.1515/almed-2026-0045 (PMC13552811; doi:10.1515/almed-2026-0045)
Supplement: Supplementary file 1 [file j_almed-2026-0045_suppl_001.docx]

**Supplementary Table 1.** Analytical and molecular characteristics of the exploratory molecular confirmation subset.

| **Patient** | **Haemoglobin (g/L)** | **Erythrocytes (x10^12^/L)** | **MCV (fL)** | **%HbS (%)** | **%HbA (%)** | **%HbA2 (%)** | **Genetic result** |
| --- | --- | --- | --- | --- | --- | --- | --- |
| 1 | 115 | 4.6 | 78 | 37.3 | 59.1 | 3.1 | 3.7 kb heterozygous deletion |
| 2 | 122 | 4.93 | 74 | 32.7 | 62.8 | 3.3 | 3.7 kb heterozygous deletion |
| 3 | 136 | 6.08 | 69 | 26.7 | 67.9 | 3.4 | 3.7 kb heterozygous deletion |
| 4 | 115 | 5.49 | 67 | 28.7 | 68 | 2.9 | 3.7 kb deletion mutation in homozygous or compound heterozygous form. |
| 5 | 125 | 4.73 | 80 | 34.3 | 60.4 | 3.4 | 3.7 kb heterozygous deletion |
| 6 | 105 | 4.87 | 70 | 27.8 | 68.3 | 3.3 | 4.2 kb heterozygous deletion |
| 7 | 142 | 5.44 | 78 | 36 | 60.3 | 3.2 | 3.7 kb heterozygous deletion |
| 8 | 109 | 4.72 | 71 | 37.9 | 58.8 | 2.8 | None |
| 9 | 117 | 4.89 | 72 | 37.7 | 59.4 | 2.4 | None |
| 10 | 124 | 4.89 | 77 | 37.9 | 58.6 | 3.2 | None |

**%HbS: percentage of haemoglobin S; %HbA: percentage of haemoglobin A; %HbA2: percentage of haemoglobin A2; %HbF: percentage of haemoglobin F; MCV: mean corpuscular volume.**
